# The dorsal posterior insula is not an island in pain but subserves a fundamental role - Response to: “Evidence against pain specificity in the dorsal posterior insula” by Davis *et al.*

**DOI:** 10.12688/f1000research.7287.1

**Published:** 2015-11-03

**Authors:** Andrew R. Segerdahl, Melvin Mezue, Thomas W. Okell, John T. Farrar, Irene Tracey

**Affiliations:** 1 Oxford Centre for Functional Magnetic Resonance Imaging of the Brain (FMRIB), Nuffield Department of Clinical Neurosciences, University of Oxford, Oxford, OX1 4BH, UK; 2 Nuffield Division of Anesthetics, Nuffield Department of Clinical Neurosciences, University of Oxford, Oxford, OX1 4BH, UK; 3 Center for Clinical Epidemiology and Biostatistics, Perelman School of Medicine, University of Pennsylvania, Philadelphia, Pennsylvania, 19104-6291, USA

**Keywords:** Pain, insula, brain imaging, ASL

## Abstract

An interesting and valuable discussion has arisen from our recent article (Segerdahl, Mezue *et al*., 2015) and we are pleased here to have the opportunity to expand on the various points we made. Equally important, we wish to correct several important misunderstandings that were made by Davis and colleagues that possibly contributed to their concerns about power when assessing our paper (e.g. actual subject numbers used in control experiment and the reality of the signal-to-noise and sampling of the multi-TI technique we employed). Here, we clarify the methods and analysis plus discuss how we interpret the data in the Brief Communication noting that the extrapolation and inferences made by Davis and colleagues are not consistent with our report or necessarily, in our opinion, what the data supports. We trust this reassures the *F1000Research* readership regarding the robustness of our results and what we actually concluded in the paper regarding their possible meaning. We are pleased, though, that Davis and colleagues have used our article to raise an important discussion around pain perception, and here offer some further insights towards that broader discussion.

We actually agree with a main premise of Davis and colleagues’ comments; namely, that the hunt for a single pain region is redundant and relying on 'single pain region' inference-based logic is flawed. The main finding presented in our study is that the contralateral dorsal posterior insula (dpIns) was the only region that was observed to track pain intensity; one of the many fundamental variables that are integrated by a number of other key brain regions into a complete tonic pain experience. At no point in the paper do we say that by identifying this ‘fundamental role for the dpIns’ in tracking pain intensity does this mean we’re “promoting the concept of a single spot”, as Davis and colleagues have themselves interpreted from our data. Nor do we ever suggest that the results presented as a Brief Communication should be used to regress back to an expired ‘one region fits all’ pedagogy of where ‘pain is in the brain’. Indeed, that view would be completely contrary to the view and concept about ‘pain representation in the brain’ that we’ve long held and have written extensively about via original studies, reviews and editorials over the past 16 years. We have even explicitly discussed the issue surrounding the concept of an ‘N1’ or ‘P1’ for pain and stated that there is ‘no P1 for pain like V1 for vision’.

The concept we’ve long held (and still do) is that due to pain being multifactorial and highly variable (even in response to the same nociceptive input) - contingent upon the context, cognitive and emotional state of an individual, it must be reflected in a malleable, flexibly accessible set of brain regions that variably activate in concert (i.e. network connectivity is key to perceptions) - (for examples, see: Berna *et al.*, 2010; Denk *et al.*, 2014; Lee & Tracey, 2013; Leknes *et al.*, 2013; Ploghaus *et al.*, 1999; Ploner *et al.*, 2010; Ploner *et al.*, 2011; Wiech *et al.*, 2008; Wiech *et al.*, 2010; Tracey, 2005a; Tracey, 2005b; Tracey, 2008; Tracey, 2011; Tracey & Johns, 2010; Tracey & Mantyh, 2007; Wiech & Tracey, 2009). Pain is not a unitary thing. Most recently, we have drawn upon computational approaches to show even that some pain experiences can be influenced by priors in a Bayesian brain - over-riding the information from nociceptive inputs to ultimately decide the pain experience (e.g. Wiech *et al.*, 2014a). Further, we have shown that even in sleeping babies an extensive network of activity subserves nociceptive processing ( Goksan *et al.*, 2015). As we’ve explicitly written many times, pain – like all perceptions - requires a network of brain activity for it to emerge. However, to identify and dissect within that complex set of brain regions (using imaging or electrophysiological methods, neurophysiological or anatomical measures) what roles different regions or signals relate to in terms of the multifactorial pain experience, as well as to identify potentially nociceptive, pain intensity or other specific features of the pain experience is what many animal and human pain researchers have done for many years. The same aim held for this study, as set out, we thought clearly, in the introduction and references quoted. For the avoidance of doubt and in the interests of absolute clarity: we hold that if a region is found to have a specific role that appears quite fundamental and important to the pain experience – whether from animal or human studies – this does not mean it’s the sole region responsible for that complex pain experience, as Davis and colleagues have concluded and suggest our data implies. That is why we used throughout the paper and in the title the expression: “subserves a fundamental role in pain” – which doesn’t mean ‘it’s the only region involved’ in pain, which we agree would be naïve bordering on incredulous. Possibly the format of a brief communication didn’t help make this position clear.

Our study explored what brain regions over a long period of time continuously activate and track a specific feature of the pain experience (in this case intensity) in response to a controlled manipulation of the nociceptive input using a novel imaging technique that allows such exploration. This is a very different approach for examining the neural correlates of painful experiences – and not one whereby the normal framework for thinking about the data can be assumed by applying what we know from conventional imaging/electrophysiological approaches and paradigms used to date (even the single-TI arterial spin labeling (ASL) studies). Perhaps this is why the highly significant result we found was so intriguing and challenging for all of us researchers to interpret, including ourselves.

Obviously no brain region, especially the insula, works in isolation as an island. Indeed, we had just showed precisely that in a publication prior to our Brief Communication; using functional and structural imaging we identified how extensively different divisions of the insula (including posterior) connect and communicate to other brain regions – even at rest ( Wiech *et al.*, 2014b). Yet, it is a fact that these other important brain regions were not continuously activated and involved in tracking a changing pain experience in the same fashion as we found was the case for the dpIns. We believe this was not for reasons of being underpowered (see below for correction to misunderstandings). In fact, even when lowering the statistical threshold dramatically, as we reported in the supplementary material of the paper but perhaps was missed, we did not find other areas significantly tracking the pain intensity ratings over the nearly two hour experiment. However, that result does not in itself nullify the relevance of these other brain regions for being necessary to bring about a complete painful experience, as we’ve shown many times (see refs above as examples). Indeed, we even showed that fact in the paper itself, as many regions were observed to have significant changes in cerebral blood flow (CBF) during the comparison of ‘peak pain’ to ‘rest’ [see: Supplementary Figure 2 in ( Segerdahl *et al.*, 2015)]. Perhaps Davis and colleagues wanted to see these supplementary data interrogated further – to see how the various regions we observed to be active during peak pain relative to baseline relate to the dpIns result? That withstanding, we’re left with how to best interpret and semantically describe our highly significant and robust result regarding the dpIns; a discussion that this platform provides a forum for.

Let us now take each criticism in turn:

**1) Experimental method**

Unfortunately, Davis and colleagues provide an incorrect assessment of the imaging modality used, the analysis tools applied and the details that are actually reported in the manuscript. The following offers important corrections and clarifications to their review:

**a. Multi-TI pCASL FMRI & absolute CBF quantification**

Advances in ASL methods over recent years have been tremendous and are actually affording widespread penetration for clinical benefit (e.g. Alsop *et al.*, 2014; Chappell *et al.*, 2012; Kelly *et al.*, 2013; Mezue *et al.*, 2014; Okell *et al.*, 2013). Davis and colleagues blur distinct, well described MRI methods and PET together into one way of 'measuring regional CBF'. Unfortunately, this ignores really important and different features of the various methods available, including the one used in our study, and the highly flexible paradigm designs that ASL based methods now afford neuroscience (compared to positron-emission tomography (PET)) to probe brain activity in very different ways and with varying degrees of improved signal-to-noise (SNR).

As we described in the paper, relative CBF (rCBF) volumes were actually collected approximately every 8s (not at 45s intervals as Davis and colleagues state). This is directly analogous to the ASL methods cited by Davis and colleagues. Further, the authors’ assertion that single-TI (or PET) is better than multi-TI ASL methods used is incorrect and unsupported when it comes to robustness of the measured blood flow and hence SNR. Rather, and as referenced in our paper, a key recognized benefit of the multi-TI approach is that it enables one to calculate absolute CBF (absCBF) at each voxel of the whole brain volume by incorporating each rCBF volume collected (i.e. at every 8s, at each TI) for the nearly two hour pain experience ( Chappell *et al.*, 2009; Mezue *et al.*, 2014; Okell *et al.*, 2013). By collecting each rCBF volume at different TIs, we are additionally able to incorporate information about changes in the time at which arterial signal arrives (i.e. arrival time = AAT) at a given voxel: a feature that is known to vary across space (i.e. all regions of the brain are not perfused instantaneously) and time (i.e. the dynamics of how perfusion changes over time during slowly-fluctuating pain is not known). This is a problem in the single-TI ASL studies quoted and accounting for this variability has been shown to maximize the robustness and reliability of measuring CBF over time within- and between-subjects (e.g. Mezue *et al.*, 2014). In short, the multi-TI ASL method we used does not suffer from reduced temporal SNR efficiency compared to single-TI ASL (or worse, PET). In fact quite the opposite; using a multi-TI approach allows the ASL signal to be sampled around its peak, improving the SNR efficiency compared to single-TI ASL methods where a long TI must be used to reduce sensitivity to arterial transit time at which point the ASL signal has decayed considerably ( Alsop *et al.*, 2014).

To illustrate the robustness of our ASL data further, consider the temporal resolution of the data collection in our study versus the study by Owen *et al.* (2010) (cited by Davis and colleagues). While our behavioural data were collected at regular intervals comparable to what was used by Owen *et al.* (2010) [i.e. 21 behavioural data points in our study vs. 25 in Owens *et al.*, 2010], in our study each ASL data point plotted in Figure 2 of the manuscript is far more rigorously sampled with more than double the number of rCBF time points being used to quantify CBF over the course of the full experimental paradigm. When Davis and colleagues assert that the study is significantly underpowered because it is only sparsely sampling brain activity once every 45s - this reflects a significant misunderstanding of the methods used in the study and the SNR benefits of our approach.

It is true that the temporal resolution of this method is longer than single-TI approaches. Any fast changes in CBF during this period will not be accurately represented in the output data. But that is not relevant here, as our experimental design was to observe brain activity linked to slowly changing sensory states that evolve over nearly two hours and were controlled by us – so the parameters of our measurement are appropriate for (indeed far faster than) the changing behavior being investigated (behavioural ratings were collected every 2.5 minutes, remember). Any variations that are occurring faster than the temporal resolution are attributed as noise in the fitting process, are represented in the variance maps that are utilized in subsequent levels of analyses and are therefore unlikely to bias the results in any significant way. We propagated all uncertainty in the CBF estimates through each stage of the analysis such that it was incorporated into the group level effects reported in the paper. In no way are we failing to observe potentially meaningful changes in CBF nor are we merely disregarding potential sources of variation that may be occurring on this time scale.

We did detail all these aspects in the relevant method sections and we were careful to highlight specific references to recent work that further details how the method is used and what the significant benefits are for such neuroscience applications. Please refer to the following references for further insight into these developments ( Chappell *et al.*, 2009; Mezue *et al.*, 2014; Okell *et al.*, 2013).

**b. FMRIB Software Library**

All FMRIB Software Library (FSL) tools used in the paper are well-validated, publically available, ubiquitously employed across a range of experimental applications in over 1000 laboratories worldwide and are highly cited and cross-referenced. All brain activity is reported as significant using standardized and accepted criteria (e.g. voxels with supra-threshold statistics registered to a Montreal Neurological Institute (MNI)-standardized brain using standard space coordinates in FSL). The challenge of localizing group mean statistical maps is common to all fMRI studies. In the case of imaging healthy adults this is easily mitigated by employing FSL tools like Boundary-Based Registration (BBR) and FMRIBs Nonlinear Image Registration Tool (FNIRT) and defining activation clusters using Mixed Effects (z>2.3, p<0.01 cluster corrected) as Segerdahl *et al.* (2015) did. It is difficult to address Davis and colleagues’ criticisms of these approaches and the validity of our result that used globally accepted criteria without discrediting decades of fMRI imaging development, analysis optimization, and of course, the various publications by many pain imaging authors who use these very same FSL tools and criteria in their own publications. We confirmed the anatomical location of our results using the Harvard-Oxford cortical atlas in FSL that reported the greatest probability of voxels within the identified cluster of activity as residing in the insula; an observation that Davis and colleagues counter by simply referencing a different probabilistic-based atlas (Juelich). We selected the Harvard-Oxford atlas because it more comprehensively and accurately maps the entire insular cortex compared to the Juelich Atlas and therefore provides consistency with mapping of activity in other regions of the insula (i.e. the mid and anterior subdivisions).

Our finding was further validated by cross-referencing with other reported studies exploring somatotopy to nociceptive inputs within this insula region and other studies in humans performing direct cortical stimulation in this region (Figure 3 in Segerdahl *et al.*, 2015). This approach is valid and arguably, we think, goes beyond a comparison with studies that define activity within gross anatomical structures (e.g. ‘insula’, ‘posterior insula’ ‘cingulate’, ‘frontal cortex’, etc.), as is the case for the references (2,3,4,5,6,12) cited within the paper by Davis and colleagues. Excellent and relevant though these studies are, it is interesting to note that the reported coordinate of peak-activities from all these studies don’t overlap with our peak coordinate and is found to be proximal to the dpIns cluster when painful stimulation is used. We should also say though that even if they did overlap it could be argued that distinct specificity might still reside within that one cluster region considering the hundreds of thousands of neurons present – this vexing problem is common to many functional imaging studies and of course was one of the original motivations for employing multivariate pattern analyses, so that specificity to different tasks within ‘one blob’ of cluster activity could be better identified (e.g. Haynes, 2015). Also, it should probably be noted that earlier work of ours comparing the spatial specificity of neurally-derived functional activation as determined from BOLD data versus ASL highlighted ASL’s pre-eminence over BOLD – the latter being biased by local draining veins in some instances ( Tjandra *et al.*, 2005).

However, Davis and colleagues do highlight a very meaningful topic of discussion about how best to map function across controversial boundary zones where two anatomically distinct regions align, as in the case of SII and dpIns. Although it was not possible to detail this important issue within the very strict word and reference limit of a Brief Communication, we did include references to discussions about the posterior insula medial operculum region (PIMO) in order to contextualize our results within the discussion regarding the possibility for nociceptive coding within a sub-region of the posterior insula (see: Craig, 2014; Evrard *et al.*, 2014; Garcia-Larrea, 2012).

**2) Control experiment**

The control experiment was completed on 12 participants (not seven as stated by Davis and colleagues), and this misreading forms another basis for their comments about the statistical power of the study. For clarity, the pain paradigm was done on 17 subjects. None of the subjects reported the vibration as painful and the group mean saliency rating for the vibration was 3.12 (s.e.m = 0.265) out of 10 [see: Supplementary Figure 1 in ( Segerdahl *et al.*, 2015)].). Power calculations suggest that a minimum of 12 subjects were necessary for group level statistical tests of absCBF data collected during a continuous sensory-motor task. This was clearly defined in both the Methods Checklist and the relevant methods sections of the paper.

However, Davis and colleagues list a few worthy points about the control experiment that we would like to discuss in more detail here. Unfortunately, there was not adequate space to fully report the extensive preliminary work we conducted in preparation for this study that explores these very issues. First, the control experiment should be a stimulus that is as relevant, captivating and as engaging as the pain but without ever being painful (or unpleasant/pleasurable) for approximately 2hrs. Obviously, the suggestion to use an innocuous warm stimulus is sensible. Unfortunately, in practice it fails to fulfill these criteria (i.e. subjects report that it is not salient and not readily perceptible after a short period of time [unpublished pilot data; Psychophysical study currently ongoing]). In this regard, vibration improves upon these limitations, as it has the salience Davis and colleagues recognize as important and is perceptible, so long as the duration of the constant stimulation is limited to the timeframe we scanned in the study. We note, even the work by Owen *et al.* (2010) suffers from having the 'control' infusion induce pain and therefore not being completely innocuous.

Therefore, it was not immediately evident how to compare pain and vibrotactile related effects directly given the nature of the experimental paradigms used.

Instead, the design was established to compare the anatomical locations of CBF changes triggered by innocuous versus noxious tonic input where saliency is closely matched. However, as Davis *et al.* correctly point out, a direct comparison of the two conditions is a standard statistical approach used in other imaging modalities and paradigms, so we agree it could be informative for this discussion. Therefore, we’ve performed a comparison of the group level effects of the correlation between CBF and intensity ratings during pain versus the correlation between CBF and intensity ratings during vibration - where all experimental variables are closely matched (i.e. 12 subjects in each group (randomly selected from the pain cohort), 14 minutes of absolute CBF data included, group mean intensity is 3 out of 10 for both modalities).

At the group level, the unpaired t-test across conditions shows that the correlation between pain and intensity ratings is localised to the contralateral dpIns (compared to the vibration-related effects); whilst a conjunction analysis confirms that there is no significant overlap between the group mean effects of either condition here (Randomise: voxelwise, p<0.05). The authors’ logic dictates that increasing the N or lengthening the vibration task to boost SNR would potentially ‘reveal’ activity related to vibration within the dpIns region. However, this logic might reversibly also predict that the subthreshold SII activity seen in the vibration results would be now shown in the pain intensity tracking result (to a suprathreshold level perhaps)- yet this does not occur even with 17 subjects and including all data points. Therefore, while we acknowledge that thermosensation might well be represented within the dpIns – ongoing work in the laboratory that we look forward to sharing (accepting the saliency issue is still a problem here) – we hope this has helped clarify the parameters used for the control task and issues surrounding its use within these more complex paradigm designs. As an aside, Davis and colleagues' reference to early PET studies of vibration (refs: 4,5) aren't precisely relevant to the current discussion as the aim of our study was to investigate unique correlates of perception not to report what the main effects of different stimulus conditions relative to baseline are.

**3) Interpretation of the data**

Davis and colleagues criticized the discussion of our findings in the light of other literature regarding insula activity, saying that we missed important references and broader discussions. Alas, we would have very much liked to include that extensive and informative literature. However, we remind Davis and colleagues (and the readership) that this was not possible within the very strict requirements for a Brief Communication where we’re only allowed 20 references and 1200 words. Further, as Davis and colleagues are aware, much of our own published work has (ironically) focused on characterizing and dissecting the roles that different insula divisions play in the multifactorial experience that is acute and chronic pain (e.g. Baumgärtner *et al.*, 2010; Brooks *et al.*, 2005; Brooks & Tracey, 2007; Duff *et al.*, 2015; Fairhurst *et al.*, 2012; Ploghaus *et al.*, 1999; Ploner *et al.*, 2010; Ploner *et al.*, 2011; Schweinhardt *et al.*, 2006; Wanigasekera *et al.*, 2012; Wiech *et al.*, 2010; Wiech *et al.*, 2014a; Wiech *et al.*, 2014b; Wise *et al.*, 2002) alongside the somatotopic studies quoted in the original paper) – nearly all of these references we couldn’t discuss or quote either due to space constraints despite their absolute relevance. Therefore, with full awareness of the literature cited alongside our own substantial contributions in understanding the complex role that the insula plays in pain mechanisms, we believe that we carefully interpreted our results while drawing upon that corpus of knowledge in the limited words allowed. We don’t believe our data undermines Craig's novel and important theories about interoception and the 'sentient self' - mechanisms that are unlikely to be anchored solely to the posterior insula (as asserted) and instead necessitate dynamic interaction with other regions such as the anterior insula, as Craig has stated ( Craig, 2015).

We agree that a more insightful discussion centers on interesting recent work that attempts to rigorously interrogate the pain specificity of statistics maps by using multivariate pattern analysis (MVPA), as we and others have been doing (see: Brodersen *et al.*, 2012; Brown *et al.*, 2011; Duff *et al.*, 2015; Marquand *et al.*, 2010; Wager, 2015; Wager *et al.*, 2013). Recent work by Woo *et al.* (2014) employed this approach to derive unique patterns of activation in regions like the dpIns that are specific to physical but not emotional pain (previously and wrongly inferred to be the ‘same’ because of overlapping BOLD statistics maps, as discussed above). Interestingly, their data support the interpretation that a region like the dpIns (see Figure 3 of Woo *et al.*, 2014) has an identifiable role in coding attributes about nociceptive driven painful experiences.

In keeping with basic principles of good experimental design, we formulated our hypothesis to test one theory about which brain regions track the intensity of a slowly varying experience of pain in response to a carefully controlled change in peripheral nociceptive input. Understanding which brain regions are coding this information is of interest, we believe. The affective coding of that stimulus is highly relevant too - processing that is likely to engage higher-level pain network processing secondary to the more initial intensity coding of the input, as nicely described in a recent review ( Garcia-Larrea & Peyron, 2013).

## Conclusion

In closing, we would like to note that imaging tonic, slowly-fluctuating and spontaneous pain states in healthy controls (and in patients) is a small, emerging field within the pain neuroimaging community with very few laboratories to date using the method. In part, this is due to the difficulty in imaging the brain during pain experiences that are analogous to what patients are suffering from, alongside limitations in accessing state-of-the-art acquisition methods for measuring blood flow using ASL. It is difficult to induce a tonic pain state in volunteers that is robust, reliable, reproducible, easily controlled and is not invasive or risks permanent skin damage or infection. A number of important ASL/pain studies have been done by colleagues and us that have laid an excellent basis for the future (e.g. Howard *et al.*, 2011; Howard *et al.*, 2012; Liu *et al.*, 2013; Owen *et al.*, 2008; Owen *et al.*, 2010; Owen *et al.*, 2012; Maleki *et al.*, 2013; Stagg *et al.*, 2013; Segerdahl *et al.*, 2012; Tracey & Johns, 2010; Tjandra *et al.*, 2005 – alongside others quoted in the paper and embedded methods references). Neither should we ignore the foundational imaging physics work done in developing quantitative cerebral perfusion imaging (i.e. ASL) over the past several decades that have provided such opportunities for clinical and basic neuroscience (e.g. Detre & Aslop, 1999; Davies & Jezzard, 2003; Figueiredo *et al.*, 2005; Guo & Wong, 2015; MacIntosh *et al.*, 2008; Mutsaerts *et al.*, 2015; Teeuwisse *et al.*, 2014; Wang *et al.*, 2003; Wong *et al.*, 2006; Wong, 2007).

We hope our response informs the *F1000Research* readership about exciting developments in the field of novel ASL applications, underscores the robustness of our paradigm and reliability of the analyses used to quantify CBF dynamics; and lastly highlights the importance of evolving conceptually away from antiquated pain imaging dogmas (derived mostly from reverse inference-based statistical testing) about single “pain spots”. In that regard, we believe Davis and colleagues agree with us. We hold that if a brain region is continuously and metabolically active in response to a slowly varying peripheral nociceptive input and further that its degree of activity correlates with a concomitant changing subjective experience of pain intensity lasting over several hours then it must be important. We can argue semantics, but it seems to us that describing it as “subserving a fundamental role in pain” (does not mean only region) and potentially being an area that might be responsive only to nociceptive inputs that produce painful experiences – i.e. nociceptive/pain specific (alongside other areas to be yet possibly identified) best describes the data. We welcome alternative interpretations or descriptions of our results, having defended their reliability. We are of course eager to interrogate and explore the meaning of this result further within ongoing preclinical and clinical studies currently underway. We look forward to sharing these results with the wider community in due course.
