# Supplementary material for: The dorsal posterior insula is not an island in pain but subserves a fundamental role - Response to: “Evidence against pain specificity in the dorsal posterior insula” by Davis et al
Source: F1000Res. 2015 Nov 3;4:1207. [Version 1] doi: 10.12688/f1000research.7287.1 (PMC4706052; doi:10.12688/f1000research.7287.1)
Supplement: Peer review history

# Peer review: Referee response for version 1

**DOI:** 10.5256/f1000research.7854.r11550

**Published:** 2015-12-15

**Authors:** Luis Garcia-Larrea

**Affiliations:** 1 NEUROPAIN Team, Lyon Neuroscience Research Center (CRNL), INSERM U1028, UMR5292, Claude Bernard University Lyon 1, Lyon, France

In their ‘response paper’, Segerdahl and colleagues endeavour to correct what they consider “important misunderstandings” made by Davis *et al.* ^1^ in their target article. Most of the points raised by Davis and colleagues are of technical nature, and question the validity of the methods used by Segerdahl *et al. *to reach their conclusions. Here Segerdahl *et al. *thoroughly describe the methods employed, correct misunderstandings, and on this basis judge that the extrapolation and inferences made by Davis and colleagues are not consistent with their target report ^2^. Also, they take the opportunity to expand on a number of conceptual points that were made.

The response is broadly divided in two parts, respectively technical and interpretative. From the technical point of view, they rebut a number of points advanced in the Davis *et al.*’s paper, in particular their critique of the multi-delay ASL technique, the excessively long sampling intervals and the small number of control subjects, all of them contributing to a supposed underpower of the experiment. Segerdahl *et al.* vigorously correct several facts that seem to have been misunderstood in the Davis *et al.*’s comment, in particular that brain MRI volumes were acquired every 8 seconds –and not 45–, and that the control experiment was completed on 12 participants –not 7 as implied in Davis *et al*. These are important particulars that could have undermined the reliability of the MRI data under question; the clarification appears appropriate, and lends support to the reliability of Segerdahl *et al.*’s original results. I am unable to enter the highly technical discussion on the relative merits of uni- or multi-TI ASL methods. While the arguments advanced by Segerdahl to defend the superiority of multi-TI ASL appear solid, I must deplore that they are mostly based on their own productions, which limits generalisation. Reproducibility of functional imaging (or rather the lack of it) *is *a crucial problem ^3^^,^^4^ and quoting external sources on multi-TI procedures would have strengthened the authors’ argument.

I support and share one of the main notions in the response of Segerdahl *et al.*, namely that they never claimed to have determined a sole ‘pain centre’, and that being *fundamental *for pain experience, as they wrote, does not equate being the *sole *region responsible for such experience. It seems clear to me that identifying a role for dorsal posterior insula (dpIns) in tracking pain intensity does not automatically promote the concept of a ‘single pain spot’. I would just add a nuance: while we have abundant experimental data of the dpIns coding properties under *physiological* conditions, this may not hold for *neuropathic *states where the dorsal insula is injured of highly deafferented, in which case the perceived intensity of pain may be coded by other areas –possibly including the insular cortex *ipsilateral *to the pain, which becomes disinhibited ^5^. Also, and probably because of extensive cortical plasticity, long-standing destruction of both posterior insulae has been shown to be compatible with the ability to continuously rate the moment-to-moment level of pain ^6^. Thus, the ‘fundamental’ role of the postero-dorsal insula tagged by Segerdahl *et al.* may be transferred to other networks in pathological conditions. 

The authors acknowledge the importance of one of the criticisms from Davis *et al.*’s comments, namely the lack of direct statistical comparison between pain and innocuous stimulation. Indeed, showing that one region exhibits significant activity changes in one condition (pain) and not in other (vibration) does not allow affirming that the activity of both regions would have differed significantly had they been compared directly. The error of comparing significance levels without directly testing their difference is said to be especially common in the neuroimaging literature ^7^^,^^8^. To respond to this criticism, now Segerdahl and colleagues performed a comparison of the correlation between CBF and intensity ratings during pain *versus *the correlation CBF / intensity ratings during vibration. This approach seems fair to me, although the formulation of the results remains ambiguous, and I remain unsure whether there was a significant difference between the two correlations, or a *region X condition* significant interaction –the straightforward result I should have expected. This could have been clarified further.

The notion of a gradient in the processing of noxious inputs from the lateral operculum (SII) to its medial portion (OP2) and posterior dorsal insula (dpIns) appears well backed by the literature. For instance, the sites where electrical stimulation induce non-noxious thermal sensations in humans predominate in the inner operculum (OP2) while those evoking clear pain tend to concentrate in the posterior insula ^9^. Also, while medial opercular sites responded to barely perceptive thermal increases, the posterior insular response only emerged clearly at noxious levels ^10^. Such a gradient is difficult to explain on a simple afferent connectivity basis, as the primate’s spinothalamic system projects in similar proportions to the granular insula and its adjacent operculum ^11^. Labelled lines entailing a significant predominance of nociceptive-specific afferents in the posterior insula might contribute an explanation ^12^, but this is difficult to reconcile with classical data showing the predominance of low threshold units in this same area ^13^^'^^14^. Alternatively, functional differences between dpIns and operculum might reflect network properties, partially independent from those of isolated neurons. For instance, the posterior insula has a more extended connectivity pattern than the opercular region and S2 proper; the massive amount of afferent input to the insula may entail a greater background activity than in the operculum, hence obstructing the precise encoding of low-energy stimuli barely emerging from background noise. This might explain why posterior insula networks are *biased toward encoding nociception* despite the fact that approximately 70% of primate insular neurons can respond to non-noxious somatic inputs too ^13^, and that intracranial recordings demonstrate posterior insular responses to non-noxious stimuli in humans ^15^. Such a bias, however, reflects a progressive gradient, and *not *a clearcut functional cleavage, and failure to acknowledge this may lead us toward dangerous neo-phrenology. The suprasylvian operculum can also encode stimulus intensity in the painful ranges ^10^, and the dpIns can also respond to non-noxious stimuli ^16^.

In conclusion, I believe the authors make clear their case that any notion of a ‘single pain centre’ in the human brain is absent from their line of thinking –and absent from the conclusions in their target paper. They also point out various methodological points that seem to have been misunderstood in the Davis *et al*.’s comment, while accepting the importance of others, such as the lack of direct statistical comparison between conditions. The important role we can ascribe to the dpIns should not be confounded with an absolute specificity of this area –especially when it comes to neuropathic lesions. And importantly, Segerdahl *et al*. acknowledge that the comments from Davis and her colleagues have triggered a most interesting, valuable and enlightening discussion, which should help many readers to get insight into current controversies on pain perception. To me, this is exactly how science advances.

I have read this submission. I believe that I have an appropriate level of expertise to confirm that it is of an acceptable scientific standard.

## References

[1] DavisKDBushnellMCIannettiGDSt LawrenceKCoghillR: Evidence against pain specificity in the dorsal posterior insula. *F1000Res*.2015;4: 10.12688/f1000research.6833.1 362 10.12688/f1000research.6833.1 26401267PMC4566284

[2] SegerdahlARMezueMOkellTWFarrarJTTraceyI: The dorsal posterior insula subserves a fundamental role in human pain. *Nat Neurosci*.2015;18(4) : 10.1038/nn.3969 499-500 10.1038/nn.3969 25751532PMC6783299

[3] BennettCMMillerMB: How reliable are the results from functional magnetic resonance imaging?. *Ann N Y Acad Sci*.2010;1191: 10.1111/j.1749-6632.2010.05446.x 133-55 10.1111/j.1749-6632.2010.05446.x 20392279

[4] ReardonS: Neuroscience in court: The painful truth. *Nature*.2015;518(7540) : 10.1038/518474a 474-6 10.1038/518474a 25719648

[5] PeyronRSchneiderFFaillenotIConversPBarralFGGarcia-LarreaLLaurentB: An fMRI study of cortical representation of mechanical allodynia in patients with neuropathic pain. *Neurology*.2004;63(10) :1838-46 1555749910.1212/01.wnl.0000144177.61125.85

[6] FeinsteinJSKhalsaSSSalomonsTVPrkachinKMFrey-LawLALeeJETranelDRudraufD: Preserved emotional awareness of pain in a patient with extensive bilateral damage to the insula, anterior cingulate, and amygdala. *Brain Struct Funct*.2015; 10.1007/s00429-014-0986-3 10.1007/s00429-014-0986-3 25577137PMC4734900

[7] PoldrackRAFletcherPCHensonRNWorsleyKJBrettMNicholsTE: Guidelines for reporting an fMRI study. *Neuroimage*.2008;40(2) : 10.1016/j.neuroimage.2007.11.048 409-14 10.1016/j.neuroimage.2007.11.048 18191585PMC2287206

[8] NieuwenhuisSForstmannBUWagenmakersEJ: Erroneous analyses of interactions in neuroscience: a problem of significance. *Nat Neurosci*.2011;14(9) : 10.1038/nn.2886 1105-7 10.1038/nn.2886 21878926

[9] MazzolaLIsnardJMauguièreF: Somatosensory and pain responses to stimulation of the second somatosensory area (SII) in humans. A comparison with SI and insular responses. *Cereb Cortex*.2006;16(7) : 10.1093/cercor/bhj038 960-8 10.1093/cercor/bhj038 16177270

[10] FrotMMagninMMauguièreFGarcia-LarreaL: Human SII and posterior insula differently encode thermal laser stimuli. *Cereb Cortex*.2007;17(3) : 10.1093/cercor/bhk007 610-20 10.1093/cercor/bhk007 16614165

[11] DumRPLevinthalDJStrickPL: The spinothalamic system targets motor and sensory areas in the cerebral cortex of monkeys. *J Neurosci*.2009;29(45) : 10.1523/JNEUROSCI.3398-09.2009 14223-35 10.1523/JNEUROSCI.3398-09.2009 19906970PMC2796243

[12] CraigAD: Topographically organized projection to posterior insular cortex from the posterior portion of the ventral medial nucleus in the long-tailed macaque monkey. *J Comp Neurol*.2014;522(1) : 10.1002/cne.23425 36-63 10.1002/cne.23425 23853108PMC4145874

[13] RobinsonCJBurtonH: Somatic submodality distribution within the second somatosensory (SII), 7b, retroinsular, postauditory, and granular insular cortical areas of M. fascicularis. *J Comp Neurol*.1980;192(1) : 10.1002/cne.901920106 93-108 10.1002/cne.901920106 7410615

[14] SchneiderRJFriedmanDPMishkinM: A modality-specific somatosensory area within the insula of the rhesus monkey. *Brain Res*.1993;621(1) :116-20 822106210.1016/0006-8993(93)90305-7

[15] PerchetCFrotMVartiainenNClaudeLGarcia-LarreaLBastujiH: A comparison of the cerebral networks activated by painful and non-painful stimulations in humans: an intra-cerebral recording study. *Paper to the 2015 European Congress of Clinical Neurophysiology*.2015;ID 304:pp. 354 Reference source

[16] BjörnsdotterMLökenLOlaussonHVallboAWessbergJ: Somatotopic organization of gentle touch processing in the posterior insular cortex. *J Neurosci*.2009;29(29) : 10.1523/JNEUROSCI.0400-09.2009 9314-20 10.1523/JNEUROSCI.0400-09.2009 19625521PMC6665561

# Peer review: Referee response for version 1

**DOI:** 10.5256/f1000research.7854.r11308

**Published:** 2015-11-25

**Authors:** Arthur Craig

**Affiliations:** 1 Atkinson Research Laboratory, Barrow Neurological Institute, Phoenix, AZ, USA

Segerdahl and colleagues have replied clearly and thoughtfully to the issues raised by Davis *et al.* regarding the short paper in Nature Neuroscience. Their response seems collegial in tone, granting trans-atlantic cultural differences (e.g., the imperative “remember” might be received less positively in America than in Britain). Further, they recognize the benefits of a constructive discussion that is open to all, rather than admonishing the commentators for overlooking key statements and details (one of which was buried near the end of the legend of Supplementary Figure 2). On the whole, though, the discussion between these two camps resembles recurring disagreements between “splitters” and “lumpers” in pain research (e.g., see Berkley, 1980) as well as, more generally, the persistent controversy about functional localization in brain research. Nevertheless, a major difference between these two sides is that one group incorporates certain knowledge from work in the monkey, which to my mind is fundamentally significant.

Davis and colleagues took issue mainly with the impression that Segerdahl *et al.* had proposed that the dorsal posterior insula (dpIns) is a “pain-specific” locus in human. That assertion is intimated in the initial portions of the paper, however the reply makes clear that **the central issue** is rather, why did the Multi-TI pCASL method reveal such “highly significant and robust” activation *only* in the dpIns after whole-brain correlation with subjective ratings of tonic “pain intensity” elicited by thermal modulation of post-capsaicin cutaneous hyperalgesia. This result is surprising because considerable prior evidence predicts that subjective feelings are associated with strong activation in the anterior insula and other sites (for review, see Ch. 7 in Craig 2015 ^2^); apparently, that’s the reason that Segerdahl *et al.* submitted the short report instead of the full-length manuscript that they could (and perhaps should) have written.

Davis *et al.* suggested that such a singular result could have resulted from faulty methodology, such as inadequate temporal sampling or underpowered statistics, but Segerdahl *et al.* convincingly rule out those two possibilities. Davis *et al.* rightfully point out that the “control” vibration results are inadequate to support a claim of pain specificity, although the “robust vibrotactile [PET] activation” they cite actually occurred in the mid-insula (see Ch. 6 in Craig 2015 ^2^). In response, Segerdahl *et al.* claim that an innocuous thermal control stimulus, though desirable, would not maintain salience; however, imaging evidence suggests that an oscillating thermal stimulus would be salient and motivating (Hua *et al.* 2005; see Ch. 2 in Craig 2015 ^2^). Davis *et al.* suggested also that inadequate anatomical registration of the ensemble of brain images could have obscured the expected activation in the anterior insula that had been observed in similar experiments by Henderson *et al.* (2007) and Owen *et al.* (2010). But, Segerdahl *et al.* rectified the misunderstanding of their anatomical protocol and explained the co-localization of the activation they observed in dpIns with the comparable results from several prior studies that had used different techniques (see Fig. 3 of the original paper). Indeed, the very precise localization of activation in the most dorsal extent of the posterior insula matches exactly the location of the terminus of ascending lamina I nociceptive-specific activity, which was demonstrated in the fundus of the superior limiting sulcus in the macaque monkey by the paper they cite (Craig 2014 ^6^; see Ch. 5 in Craig 2015 ^2^). Further, the antero-posterior somatotopic gradient identified in the monkey fits with the topographic order reported in the studies that Segerdahl *et al.* collated in Fig.3 and also with the posterior location of the “very specific ‘spot’” that they found.

Lastly, Davis *et al.* challenged the interpretation of any activation locus as “pain-specific” and suggested that “the dpIns likely is involved in pain but overall is a non-specific perceptual way-station,” thereby ignoring the evidence in the monkey. Segerdahl *et al.* naturally replied that the evidence regarding multifactorial pain-related activation includes their “own substantial contributions,” meanwhile acknowledging the new conceptual contributions by the Multi-Voxel Pattern Analysis (MVPA) study of Wager *et al.* (2013) and by the correlative functional connectivity patterns found in individual subjects by Cheng *et al.* (2015) (from Davis’ lab). Yet, on one hand they cite attributions of higher-level cognitive and affective coding to the anterior insula in reviews by Garcia-Larrea & Peyron (2013) and Craig (2015 ^2^), and on the other they point out that the new MVPA findings “support the interpretation that a region like the dpIns (see Figure 3 of Woo *et al*. 2014) has an identifiable role in coding attributes about nociceptive driven painful experiences.” Hmm. Does that mean modality-specific? noci-specific? algosity (see Greenspan’s comments)? a “pain switch”? or perhaps, an embedded cluster of neurons? They cannot say. Instead, Segerdahl *et al.* reiterate and expand the carefully worded concluding statements of their original paper, saying: “A growing body of literature suggests that a subsection of the posterior insula is both anatomically and functionally well suited to serve a primary and fundamental role in pain processing.” And “Using … a newly developed procedure and analysis, we were able to identify the dpIns as subserving a fundamental role in pain and the likely human homolog of the nociceptive region identified from animal studies. Future work targeting dpIns activity might provide a window to explore fundamental mechanisms related to how pain emerges from nociception as well.” These ideas and words remind me of Chapters 5 and 6 of my book and I must certainly agree.

OK, so far, so good. Yet, the central issue remains: why *did* their rigorous quantitative analysis demonstrate robust activation *only* in the contralateral dpIns? Now, I will make a suggestion.

The published evidence in the monkey tells us that a somatotopically-organized region of posterolateral thalamus that contains only nociceptive neurons projects topographically to the dpIns (Craig, 2015 ^2^; 2014 ^6^). The thalamic region includes both nociceptive-specific (NS) and polymodal nociceptive (HPC for heat, pinch, and cold) neurons that receive monosynaptic input from lamina I spinothalamic neurons; an adjacent sub-region of thalamus similarly relays thermoreceptive-specific (COOL) activity to an adjacent sub-region of the dpIns (as depicted in Fig. 12 in Craig 2015 ^2^). Both classes of nociceptive neurons in the monkey respond to topical capsaicin and become sensitized, with trajectories that parallel human pain reports (unpublished obs.). The HPC neurons constitute the only sensory channel that can quantitatively explain human reports of temporally summating second (burning) pain in response to repeated brief-contact heat stimulation (Craig, 2004) as well as pain reports in response to the thermal grill (Ch. 3 in Craig, 2015 ^2^); we can posit that they are directly responsible for the pain reports in the study of Segerdahl *et al.* Thus, the terminus of the HPC sensory channel in the dpIns could alone explain the activation focus they observed. Particular components of the parallel NS (first, sharp pain) sensory channel most likely would also be involved.

However, the anomalous innocuous cold sensitivity of HPC neurons and the evidence for the involvement of some HPC neurons in signaling muscle work, vascular distension, and tissue immune and metabolic conditions all indicate that this sensory channel is *not* a binary, pain-specific signal but rather has a much broader role in interoception (Craig 2015 ^2^). Along with sensory channels representing many other specific and non-specific bodily sensations, such as cool, warm, itch, sensual touch, taste, hunger, and thirst, the dpIns is thus proposed to contain *interoceptive cortex*. Both Davis *et al.* and Segerdahl *et al.* alluded to this perspective and also cited the evidence for thermoreceptive-specific activity in the dpIns, but Davis *et al.* seemingly regarded this knowledge as support for the assertion that the dpIns does not have a specific role in pain, which is a misrepresentation.

Yet, these considerations do not resolve the central issue. For instance, the HPC channel is also relayed to area 3a in the primary sensorimotor area (Vierck *et al.,* 2013; Craig, 2015 ^2^; 2014 ^6^), but that region was not activated. More importantly, neither the mid-insula nor the anterior insula displayed activation, and both are normally associated with subjective reports of immediate feelings of pain.

To my mind, the key lies in the instructions to the individuals who participated in the experiment of Segerdahl *et al*., which can suggest what their brains were doing. Unfortunately, the exact instructions were not reported; we’re told only that they were asked to report the “pain intensity” at certain times and otherwise to remain focused on a fixation cross. We can surmise that, in order to perform as requested (and presumably earn a monetary reward), the participants (1) concentrated their attention on the intensity of sensation that originated from the stimulus site, but otherwise (2) ignored the ongoing feeling of unpleasantness as much as possible. Our brains are rather good at both tasks; first, we regard pain as a discriminative sensory capacity *because* we can focus our attention on the specific characteristics and the specific origin of the sensation, and second, as a homeostatic emotion that is crucial for survival, like hunger, pain has top motivational priority most of the time but can be inhibited when other emotional needs or goals are more urgent. Based on the available evidence for the neural correlates of focused attention and emotional feelings, that suggests (1) that focal activity at the somatotopically appropriate site in the HPC map within interoceptive cortex was enhanced by the center-surround (sombrero-like) modulation of endogenous attentional processes and (2) that activity that normally engenders the feeling of unpleasantness or pain was suppressed in interoceptive integration mechanisms in the mid-insula and anterior insula (and in related regions, such as area 3a and area 24c in the anterior mid-cingulate).

To my mind, the mechanisms I propose offer a potential explanation for the singular observations of Segerdahl *et al*. They are incomplete – for instance, focal activation was not reported in the posterolateral thalamus and activity supporting the behavior of reporting the discriminative readout is missing – nonetheless, they provide practical inroads for future experiments. Most importantly, they incorporate knowledge about the crucial and specific role of dpIns in nociception and pain, and they build on the significant advance that the work of Segerdahl *et al*. has provided.

I have read this submission. I believe that I have an appropriate level of expertise to confirm that it is of an acceptable scientific standard.

## References

[1] BerkleyKJ: Spatial relationships between the terminations of somatic sensory and motor pathways in the rostral brainstem of cats and monkeys. I. Ascending somatic sensory inputs to lateral diencephalon. *J Comp Neurol*.1980;193(1) : 10.1002/cne.901930119 283-317 10.1002/cne.901930119 7430431

[2] Craig AD: How Do You Feel? An Interoceptive Moment with Your Neurobiological Self.2015;Princeton University Press(Princeton) :

[3] Huale HStrigoIABaxterLCJohnsonSCCraigAD: Anteroposterior somatotopy of innocuous cooling activation focus in human dorsal posterior insular cortex. *Am J Physiol Regul Integr Comp Physiol*.2005;289(2) : 10.1152/ajpregu.00123.2005 R319-R325 10.1152/ajpregu.00123.2005 15805097

[4] HendersonLAGandeviaSCMacefieldVG: Somatotopic organization of the processing of muscle and cutaneous pain in the left and right insula cortex: a single-trial fMRI study. *Pain*.2007;128(1-2) : 10.1016/j.pain.2006.08.013 20-30 10.1016/j.pain.2006.08.013 17011704

[5] OwenDGClarkeCFBureauYGanapathySPratoFSSt LawrenceKS: Measuring the neural response to continuous intramuscular infusion of hypertonic saline by perfusion MRI. *J Magn Reson Imaging*.2012;35(3) : 10.1002/jmri.22814 669-77 10.1002/jmri.22814 21953816

[6] CraigAD: Topographically organized projection to posterior insular cortex from the posterior portion of the ventral medial nucleus in the long-tailed macaque monkey. *J Comp Neurol*.2014;522(1) : 10.1002/cne.23425 36-63 10.1002/cne.23425 23853108PMC4145874

[7] WagerTDAtlasLYLindquistMARoyMWooCWKrossE: An fMRI-based neurologic signature of physical pain. *N Engl J Med*.2013;368(15) : 10.1056/NEJMoa1204471 1388-97 10.1056/NEJMoa1204471 23574118PMC3691100

[8] ChengJCErpeldingNKucyiADeSouzaDDDavisKD: Individual Differences in Temporal Summation of Pain Reflect Pronociceptive and Antinociceptive Brain Structure and Function. *J Neurosci*.2015;35(26) : 10.1523/JNEUROSCI.5039-14.2015 9689-700 10.1523/JNEUROSCI.5039-14.2015 26134651PMC6605146

[9] Garcia-LarreaLPeyronR: Pain matrices and neuropathic pain matrices: a review. *Pain*.2013;154 Suppl 1: 10.1016/j.pain.2013.09.001 S29-43 10.1016/j.pain.2013.09.001 24021862

[10] CraigAD: Lamina I, but not lamina V, spinothalamic neurons exhibit responses that correspond with burning pain. *J Neurophysiol*.2004;92(4) : 10.1152/jn.00385.2004 2604-9 10.1152/jn.00385.2004 15163673

[11] VierckCJWhitselBLFavorovOVBrownAWTommerdahlM: Role of primary somatosensory cortex in the coding of pain. *Pain*.2013;154(3) : 10.1016/j.pain.2012.10.021 334-44 10.1016/j.pain.2012.10.021 23245864PMC4501501
